# Severe hemoperitoneum resulting from restart of letrozole after oocyte retrieval procedure: a case report

**DOI:** 10.1186/s13256-021-02938-8

**Published:** 2021-06-27

**Authors:** Haipeng Huang, Yasushi Takai, Kouki Samejima, Yosuke Gomi, Tatsuya Narita, Shunichiro Ichinose, Yukiko Itaya, Yosihisa Ono, Sigetaka Matsunaga, Masahiro Saitoh, Hiroyuki Seki

**Affiliations:** Department of Obstetrics and Gynecology, Saitama Medical Center, Saitama Medical University, 1981 Kamoda, Kawagoe City, Saitama 350-3550 Japan

**Keywords:** Breast cancer, Fertility preservation, Hemoperitoneum, Laparoscopic surgery, Letrozole, Ovarian hemorrhage

## Abstract

**Background:**

In the field of oncofertility, patients with breast cancer are often administered letrozole as an adjuvant drug before and after oocyte retrieval to prevent an increase in circulating estradiol.

**Case presentation:**

We report a case of abdominal hemorrhage due to an ovarian rupture in a 29-year-old Japanese patient who restarted letrozole 2 days after an oocyte retrieval procedure in which 14 mature oocytes were retrieved. The patient had sought embryo cryopreservation as a fertility preservation option before undergoing treatment for recurrent breast cancer. A day after restarting letrozole treatment, the patient unexpectedly developed severe abdominal pain. Laparoscopic hemostasis was performed to manage the ovarian swelling and hemorrhage.

**Conclusions:**

The ovaries can be restimulated by restart letrozole after an oocyte retrieval procedure. Therefore, reproductive-medicine practitioners should understand the potential complications of letrozole administration in such cases and take steps to ensure that they are minimized.

## Background

Incidence of breast cancer in adolescent and young adult women is on the rise both in Japan and worldwide [1, 2]. It is also the most common disease for which patients seek assisted reproductive technology procedures for fertility preservation. In the case of hormone-dependent tumors, the use of the aromatase inhibitor, letrozole, as an adjuvant is recommended to prevent an increase in the circulating concentration of estradiol (E2) [3]. Moreover, Rodgers *et al*. concluded that letrozole does not diminish total oocyte yield [4]. Current recommendations for fertility preservation in women with E2-responsive breast cancer advise a second course of letrozole after oocyte retrieval to keep serum E2 levels low [5]. This regimen is known to significantly reduce the circulating concentration of E2 after oocyte retrieval [6]. However, letrozole has also been reported to have ovary-stimulating effects, even if administered in the luteal phase of the menstrual cycle [7]. Here, we report a case of abdominal hemorrhage due to ovarian rupture in a patient who restarted letrozole after an oocyte retrieval procedure.

## Case presentation

The patient was a 29-year-old nulligravid Japanese woman with no history of infertility, who was seeking fertility preservation in advance of chemotherapy for breast cancer that developed shortly after her marriage. Her body mass index (BMI) was 19.4 (height 157 cm, weight 48 kg). Although she had a history of asthma, she had been incident-free for more than 2 years. She had been diagnosed with breast cancer 3 years previously and was treated by partial resection of the left mammary gland. The excised mass had a positive margin and was identified as ductal carcinoma *in situ* based on histopathological findings: Estrogen receptor (ER) (+), Progesterone receptor (PR) (+), HER2 (1+), and Ki67 (10%). These findings prompted the doctors to perform a left total mastectomy, which yielded an excised mass with a negative margin. The patient tested negative for lymph-node metastasis. She declined postoperative chemotherapy and radiation therapy because of a strong desire to bear children. Follow-up tests every 3 months revealed no evidence of disease until 31 months after the total mastectomy, when subcutaneous recurrence was discovered in the left chest. The mass was surgically removed, and identified as an invasive ductal carcinoma based on histopathological findings: Nottingham Grade (NG) 1, ER (8) PR (8) HER2 (2+) fluorescence *in situ* hybridization (FISH) (2.54) Ki67 (10–20%). The patient was then scheduled for 6 months of chemotherapy consisting of cyclophosphamide and HER2-targeted therapy and trastuzumab starting roughly 2 months after tumor excision. Chemotherapy was followed by hormone therapy for a minimum of 3 years. The patient was referred to Saitama Medical Center after expressing the desire, during counseling, to preserve her fertility prior to starting chemotherapy.

The patient’s family history of multiple breast cancer reported for both her mother and grandmother led us to suspect hereditary breast and ovarian cancer syndrome, and therefore, she was referred to a genetic counselor before beginning any fertility treatment. Blastocyst cryopreservation was selected as the treatment option for fertility preservation after the ovaries were verified to be cancer-free.

Her blood tests showed normal levels of infertility-related biomarkers [anti-Müllerian hormone (AMH): 2.90 ng/mL, Base E2: 42.7 pg/mL, follicle-stimulating hormone (FSH): 5.8 mU/mL, luteinizing hormone (LH): 4.1 mU/mL] and no signs of coagulation abnormalities. Her menstrual cycle was 30 days. Her ultrasound did not reveal any characteristics of polycystic ovary syndrome. Her Pap test result was negative for intraepithelial lesions or malignancy (NILM) according to the Bethesda classification. The results of her husband’s semen analysis were normal (semen volume: 3.8 mL, sperm count: 43 million, motility: 69%).

The woman commenced oral letrozole (5.0 mg/day) on day 5 of her cycle, and started self-injecting recombinant FSH (225 IU/day) the next day. As expected, her circulating hormone levels were slightly elevated 6 days after FSH administration, [E2: 469 pg/mL, LH: 3.1 mIU/m, FSH: 19.3 mU/mL, progesterone (P4): 0.54 ng/mL], and the dominant follicle had a diameter of 16 mm. Daily administration of ganirelix (0.25 mg/day) was started on the same day. After 11 days of FSH administration, the dominant follicle had increased in size to 22 mm, and hormone levels were further elevated (E2: 1550 pg/mL, LH: 1.6 mIU/m, FSH: 16.4 mU/mL, P4: 2.23 ng/mL). At this point, we decided to proceed with the oocyte retrieval, expecting to harvest 16 eggs of suitable size (> 14 mm). Final oocyte maturation was triggered by administering two 300 μg doses of a gonadotropin releasing hormone (GnRH) agonist 1 hour apart (buserelin nasal spray 0.2 mg, Nasanyl; Pfizer, Tokyo, Japan). After 36 hours, 15 oocytes were retrieved, including 14 mature metaphase II (MII) eggs and one immature germinal vesicle (GV). The MII oocytes were fertilized before cryopreservation [split insemination: seven *in vitro* fertilization (IVF), seven intracytoplasmic sperm injection (ICSI)], preserving only the good-quality embryos; the lone GV oocyte was cryopreserved unfertilized.

**Fig. 1:**
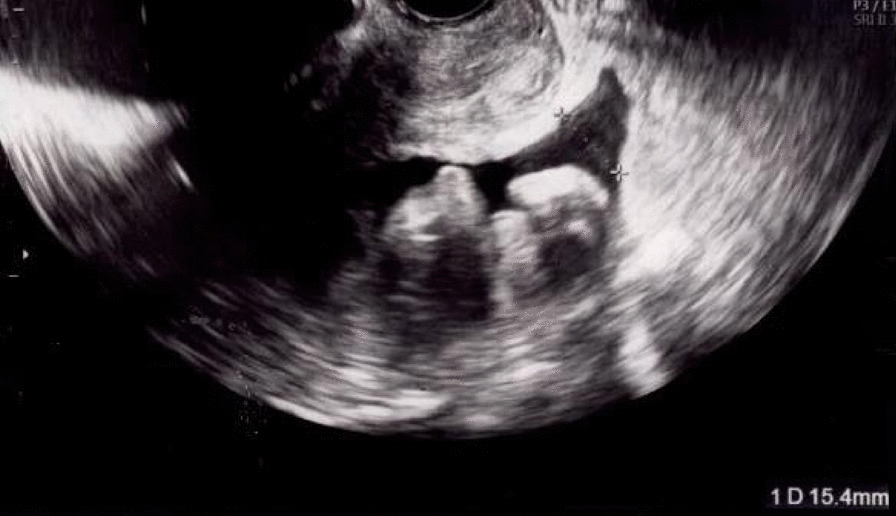
Ultrasonography showing mild ascites 2 days after oocyte retrieval

**Fig. 2:**
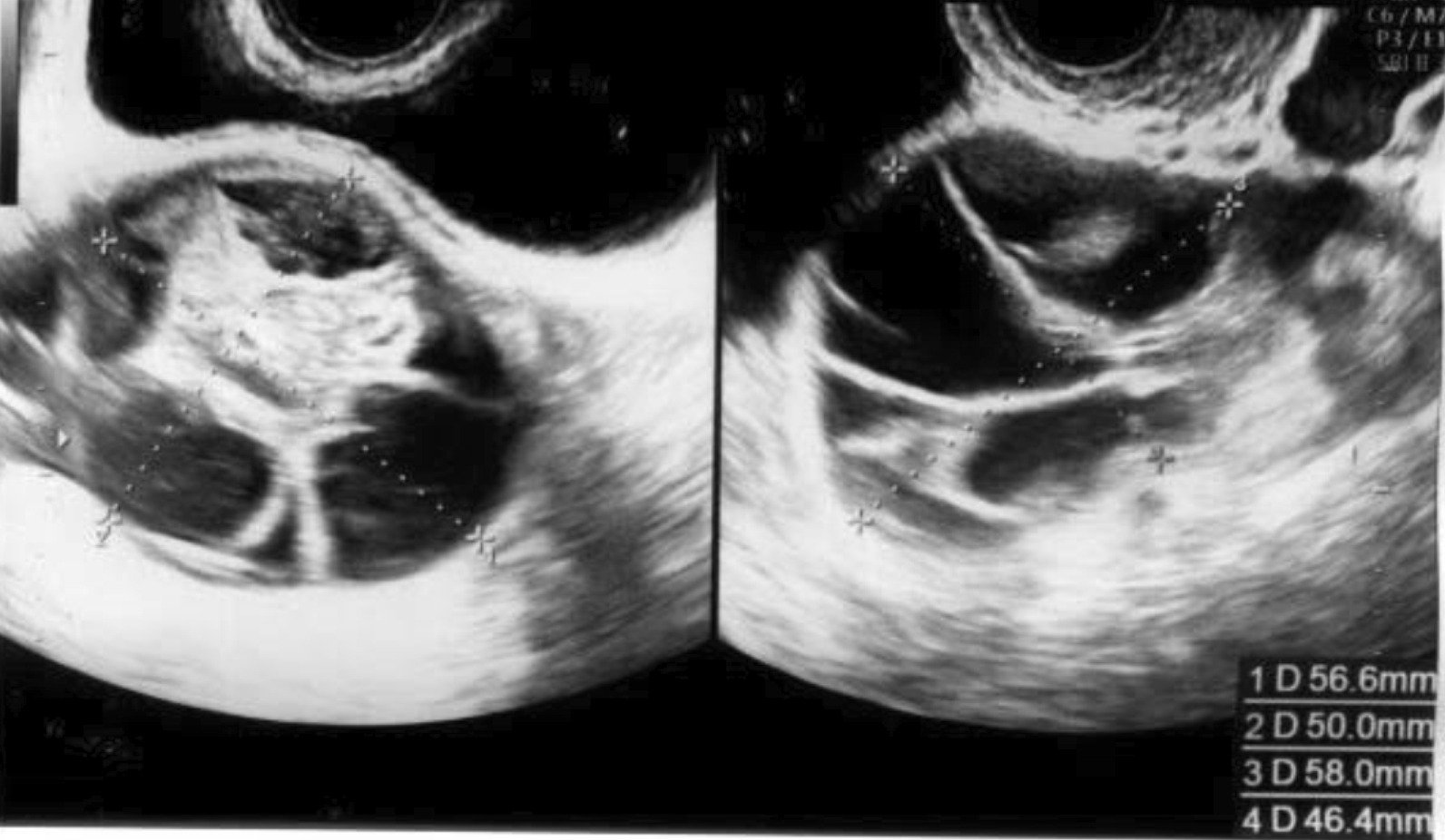
Ovarian ultrasonography showing mild ovarian swelling 2 days after oocyte retrieval

To prevent any delays in her cancer treatment due to ovarian hyperstimulation syndrome (OHSS), the patient started taking oral cabergoline (0.5 mg) after oocyte retrieval. Only mild ovarian swelling (right: 57 × 50 mm, left: 58 × 46 mm) and mild ascites were observed on a follow-up visit 2 days later, after 52 hours of the oocyte retrieval procedure (Figs. 1, 2). She did not complain of tenderness during the pelvic examination. As the patient experienced only minor abdominal pain after oocyte retrieval, analgesic use was considered unnecessary. On the same day, she was restarted on letrozole (5.0 mg/day) to prevent the increase of circulating E2, since her tumor was E2 receptor-positive. The next day, 76 hours after the oocyte retrieval procedure, she unexpectedly developed severe abdominal pain and was urgently admitted to our hospital.

**Fig. 3:**
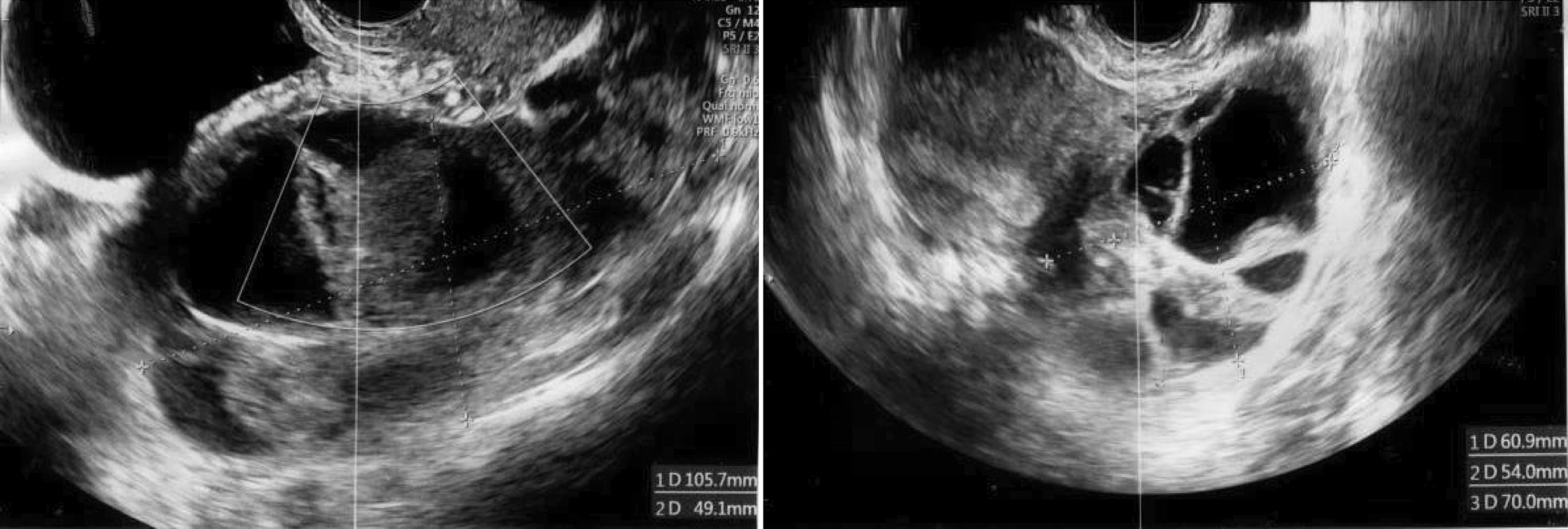
Ovarian ultrasonography 3 days after oocyte retrieval (1 day after restarted letrozole). The ultrasonograph shows bilateral exacerbation of ovarian swelling and increased ascitic volume

On admission, the patient complained of abdominal distension and lower abdominal and lower back pain. Her blood biochemistry profile was as follows: white blood cell (WBC): 8200/μL, hemoglobin (Hb): 14.2 g/dL, hematocrit (Hct): 40.1%, C-reactive protein (CRP): 0.02 mg/dL, total protein (TP): 7.2 g/dL, albumin (Alb): 4.5 g/dL, Creatinine (Cre): 0.53 mg/dL, Uric Acid (UA): 4.8 mg/dL, activated partial thromboplastin time (APTT): 31.7 seconds, prothrombin time (PT): 12.0 seconds, PT%: 107, D-dimer: 0.78 μg/mL. Ultrasonography revealed bilateral exacerbation of ovarian swelling (right: 105 × 49 mm, left: 70 × 60 mm;) and increased (moderate) ascitic volume (Fig. 3). Ovarian torsion was suspected based on tenderness and swelling noted in the left ovary.

**Fig. 4:**
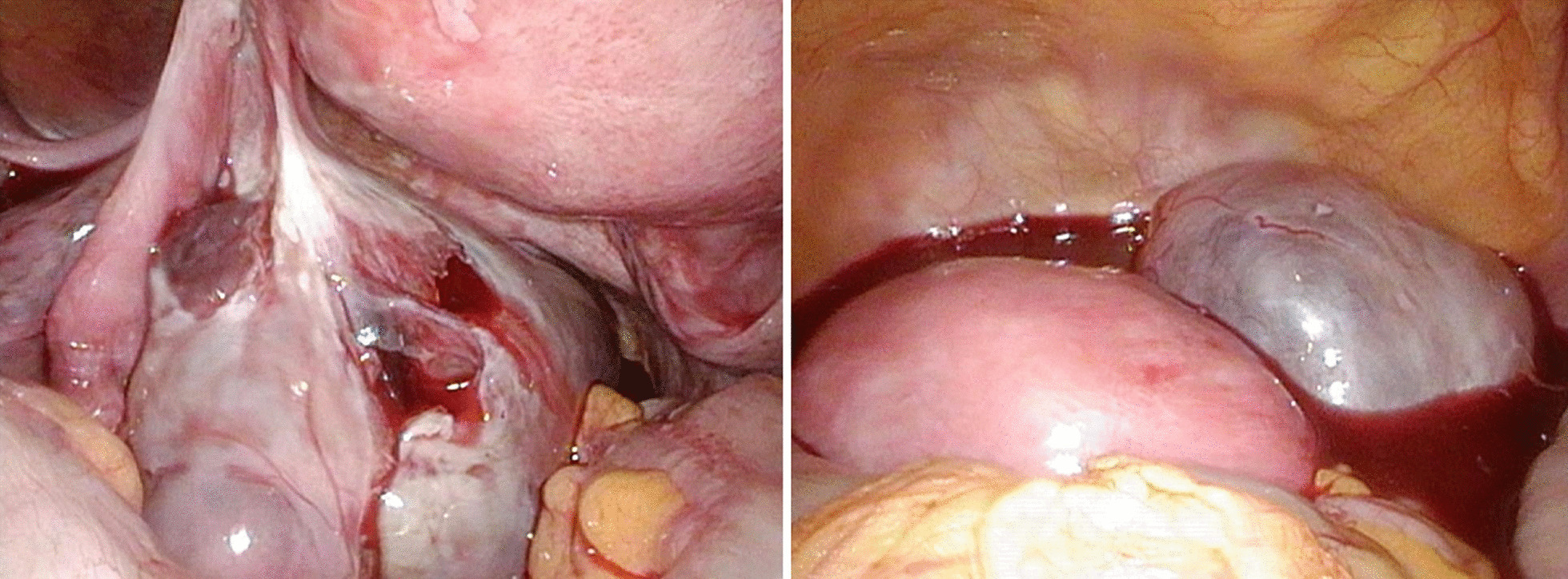
Intraoperative findings. Left: ruptured ovary; right: blood pooling in the pelvic cavity

**Fig. 5:**
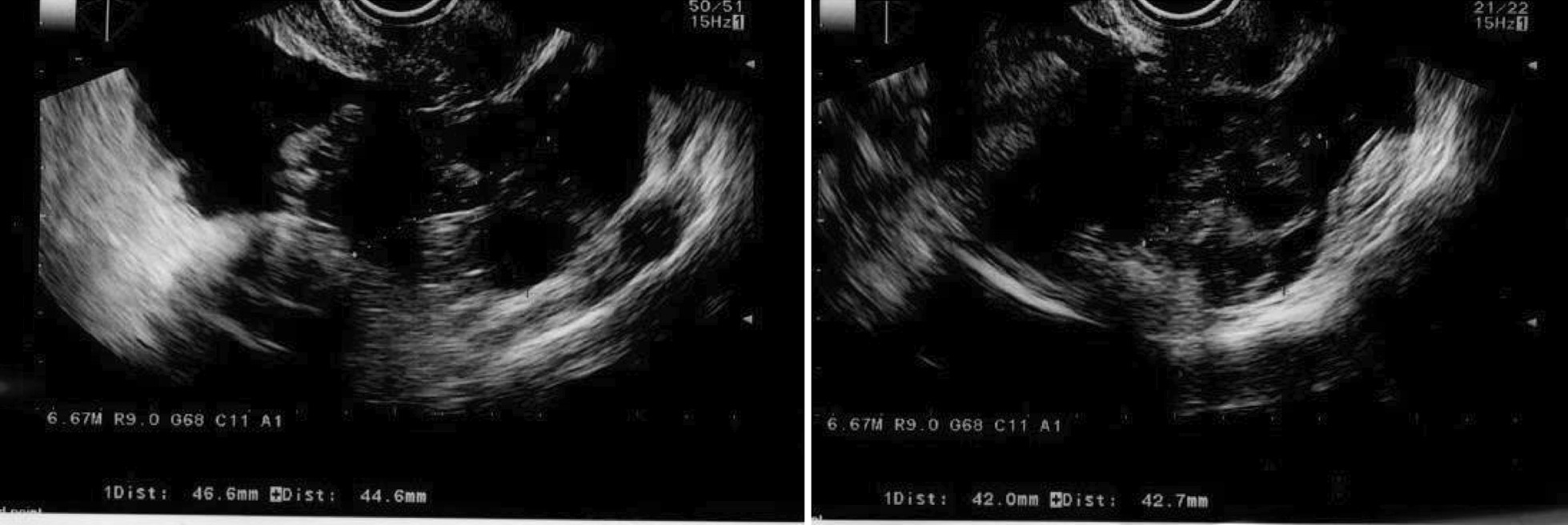
Ovarian ultrasonography before discharge, showing bilateral shrinkage of the ovarian swelling to ~ 4.5 cm size

An emergency laparoscopic surgery was performed to confirm the diagnosis. The left ovary was enlarged and had ruptured at what was apparently an aspiration site from oocyte retrieval. Hemoperitoneum, caused by continued abnormal bleeding from the same site, was also observed (Fig. 4). Laparoscopic hemostasis was performed. A day later, her blood biochemistry profile showed normal findings with a slight reduction in hemoglobin (WBC: 9400/μL, Hb: 12.7 g/dL, Hct: 36.4%, TP: 6.3 g/dL, Alb: 3.9 g/dL, Cre: 0.48 mg/dL, APTT: 30.7 seconds, PT: 12.9 seconds, PT%: 93). The patient’s general condition after the intervention was good. She was discharged from the hospital 4 days later (that is, 7 days after oocyte retrieval) after bilateral shrinkage of the ovarian swelling was confirmed by ultrasonography (right: 46 × 44 mm, left: 42 × 42 mm; Fig. 5), and no worsening of OHSS symptoms in the past week of daily oral cabergoline (0.5 mg) was noted.

## Discussion and conclusions

The incidence of severe hemoperitoneum was 0.08% in 2007, decreasing 0.29-fold by 2015 in Japan [8]. Severe hemoperitoneum symptoms usually appear within 24 hours after oocyte retrieval procedures [9].

Letrozole is an oral medication frequently used in the field of oncofertility, and few reports of complications or side effects have been published. Despite showing no signs of abnormality during a follow-up examination 2 days, that is, 52 hours, after the oocyte retrieval procedure, our patient unexpectedly experienced ovarian swelling after 76 hours of the oocyte retrieval procedure within 24 hours of restarting letrozole, which apparently caused ovarian rupture and persistent ovarian hemorrhage. Given the clinical findings and time line, the ovulation-stimulating effects of letrozole clearly contributed to the swelling observed [7]. However, as ovarian swelling is also a hallmark symptom of OHSS, this syndrome may also have played a role. We believe this case serves as a timely warning that should encourage practitioners to exercise caution when considering letrozole administration after oocyte retrieval.

Adjuvant letrozole is effective in preventing OHSS [10, 11]. In one randomized clinical trial [12] either aspirin or letrozole was taken orally for 5 consecutive days after ovulation was induced using an hCG trigger, and letrozole resulted in a lower incidence of OHSS compared with aspirin. However, 25% of the letrozole group experienced moderate or higher OHSS, and, just as concerning, patients treated with letrozole had higher serum vascular endothelial growth factor (VEGF) levels than those not treated with it. Similar research has shown increased levels of VEGF and decreased levels of pigment epithelium-derived factor (a potent angiogenesis inhibitor) in the follicular fluid of patients treated with letrozole [13]. Factors such as these could have led to the abnormal ovarian bleeding observed in our patient in the days following oocyte retrieval.

The adjuvant use of letrozole after oocyte retrieval suppresses circulating E2 and may reduce the likelihood of OHSS. Nonetheless, our experience here suggests that letrozole treatment can also lead to ovarian restimulation, causing ovarian rupture and persistent ovarian hemorrhage.

In conclusion, the use of letrozole has become essential to oocyte retrieval protocols for E2-receptor-positive breast-cancer patients seeking fertility preservation. This case report highlights a potential complication associated with letrozole treatment in such patients. Therefore, when considering letrozole administration in anticipation of subsequent oncotherapy, reproductive medicine practitioners should consider its potential complications and take appropriate steps to ensure that they are minimized.

## Data Availability

Not applicable.

## Abbreviations

E2: Estradiol
NILM: Negative for intraepithelial lesions or malignancy
MII: Metaphase II
GV: Germinal vesicle
OHSS: Ovarian hyperstimulation syndrome
VEGF: Vascular endothelial growth factor
